# Post-cardiac Injury Syndrome in a Systemic Lupus Erythematosus Patient After an Open-Heart Operation: A Case Report

**DOI:** 10.7759/cureus.46077

**Published:** 2023-09-27

**Authors:** Aseel Abuhammad, Osayd Nassar, Saed I Atawnah

**Affiliations:** 1 Faculty of Medicine, Al-Quds University, Jerusalem, PSE; 2 Internal Medicine, Al-Ahli Hospital, Hebron, PSE

**Keywords:** heart injuries, open-heart operations, case report, coronary artery bypass grafting, post-cardiac injury syndrome, systemic lupus erythematosus

## Abstract

Post-cardiac injury syndrome (PCIS) is an inflammatory condition caused by a cardiac injury that can affect the pericardium, pleura, or both. We describe a female patient who underwent heart surgery and thereafter developed pericardium and pleural effusion. She was also known to have systemic lupus erythematosus (SLE). Due to the possibility that each of these symptoms could impact the pericardium or pleura, we came to the conclusion that they could be caused by either PCIS, SLE, or both.

A 54-year-old woman underwent open heart surgery three weeks ago and required aortic valve replacement and coronary artery bypass grafting (CABG). She presented to the emergency room complaining of fatigue, chest pain, shortness of breath, coughing, and fever for five days. She had a history of SLE for seven years. The patient was found to have a right-side pleural effusion, a pericardial effusion, and a high inflammatory marker based on imaging and laboratory evaluations. A right pleural-side image-guided percutaneous pigtail catheter drainage was inserted. Exudative fluid appeared in the pleural fluid analysis, and a mild pericardial effusion was seen on echocardiography. The patient was diagnosed with pericarditis and treated with prednisone, colchicine, and antibiotics. Six days later, she was discharged in good general condition.

In this particular case of SLE with a chronic inflammatory reaction, PCIS developed following valve replacement surgery. The activation and destruction of endothelial cells are frequently seen in both SLE and PCIS, leading us to believe that there may be a possible reciprocal interaction between these two distinct autoimmune illnesses.

## Introduction

Post-cardiac injury syndrome (PCIS), commonly referred to as post-cardiac injury syndrome, is an inflammatory condition that affects the pleura, pericardium, and lung parenchyma after different types of heart surgeries or injuries and is characterized by fever, chest discomfort, tachycardia, pericardial effusion, and exudative pleural effusion [1]. A definitive diagnosis of PCIS is made with clinical suspicion and the exclusion of other differential diagnoses, including pulmonary embolism (PE), lung infection, and heart failure [1]. Despite the fact that the disease's pathogenesis is still not fully understood, it is thought to be connected to autoimmune pathogenesis, particularly in light of the rise in anti-cardiomyocyte antibodies [2]. Here, we discuss the case of a woman who underwent open-heart surgery and developed pericardial effusion, right-side pleural effusion, and high inflammatory markers. These symptoms may have been carried on by a concomitant condition, especially an autoimmune disorder or PCIS. In light of the existence of systemic lupus erythematosus (SLE), our case offers more information about the etiopathogenesis of this syndrome.

## Case presentation

A 54-year-old woman underwent open heart surgery three weeks ago and required aortic valve replacement and coronary artery bypass grafting (CABG). She presented to the emergency room complaining of fatigue, stabbing chest pain that got worse with breathing, new-onset shortness of breath, coughing, and fever for five days. She had previously been diagnosed with SLE seven years ago and had been on hydroxychloroquine 200 mg and prednisone 5 mg, increased to a high dose due to her active disease before her surgery.

**Figure 1:**
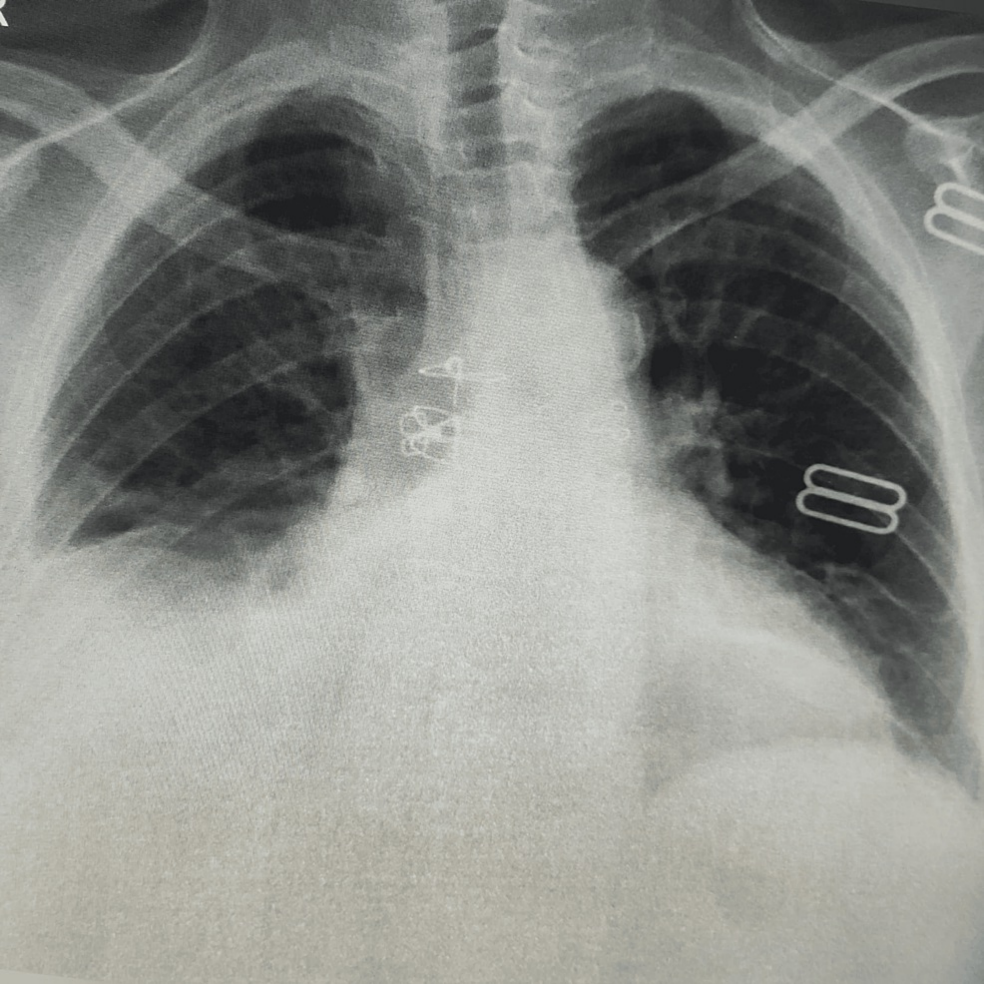
Chest X-ray on admission showing right pleural effusion

The examination was notable for the patient's 100 bpm heart rate, normal blood pressure, normal oxygen saturation, and temperature of 98.6 °F. Except for a diminished breathing sound to the right of the lung, the rest of the physical examination was unremarkable. A right-side pleural effusion was noted on a chest X-ray (Figure 1). The outcome of the laboratory investigation revealed a normal white blood cell count, normocytic anemia, normal procalcitonin, raised C-reactive protein (CRP), and high erythrocyte sedimentation rate (ESR).

**Figure 2:**
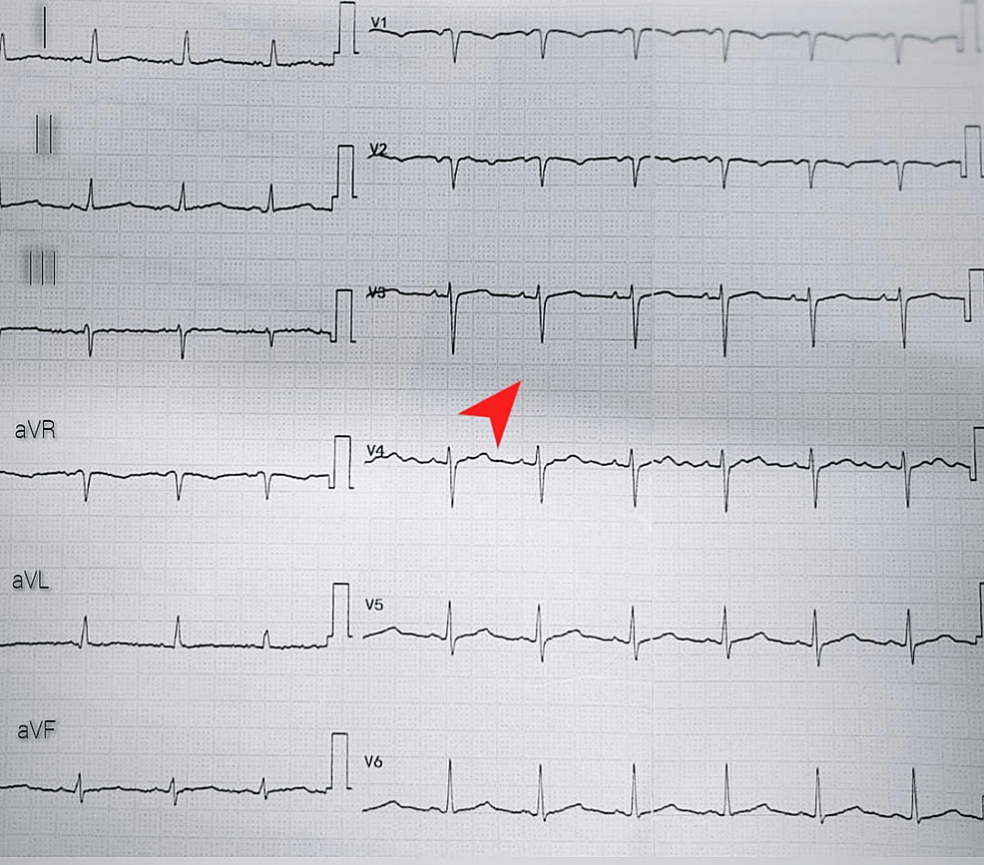
Electrocardiogram on presentation: poor R-wave progression and low voltage (red arrow)

Blood cultures and other septic workups showed negative results. Image-guided percutaneous pigtail catheter drainage was performed on the right pleural side without complications. Analyses of the pleural fluid indicated exudative fluid, and echocardiography showed mild pericardial effusion. A cardiologist made the diagnosis of pericarditis based on the clinical symptoms and echocardiography; the electrocardiogram (ECG) revealed no substantial abnormalities other than electrical alternans (Figure 2). It was noted to have pericardial effusion, pericarditis, right-side pleural effusion, and elevated inflammatory markers based on imaging and a laboratory assessment.

**Figure 3:**
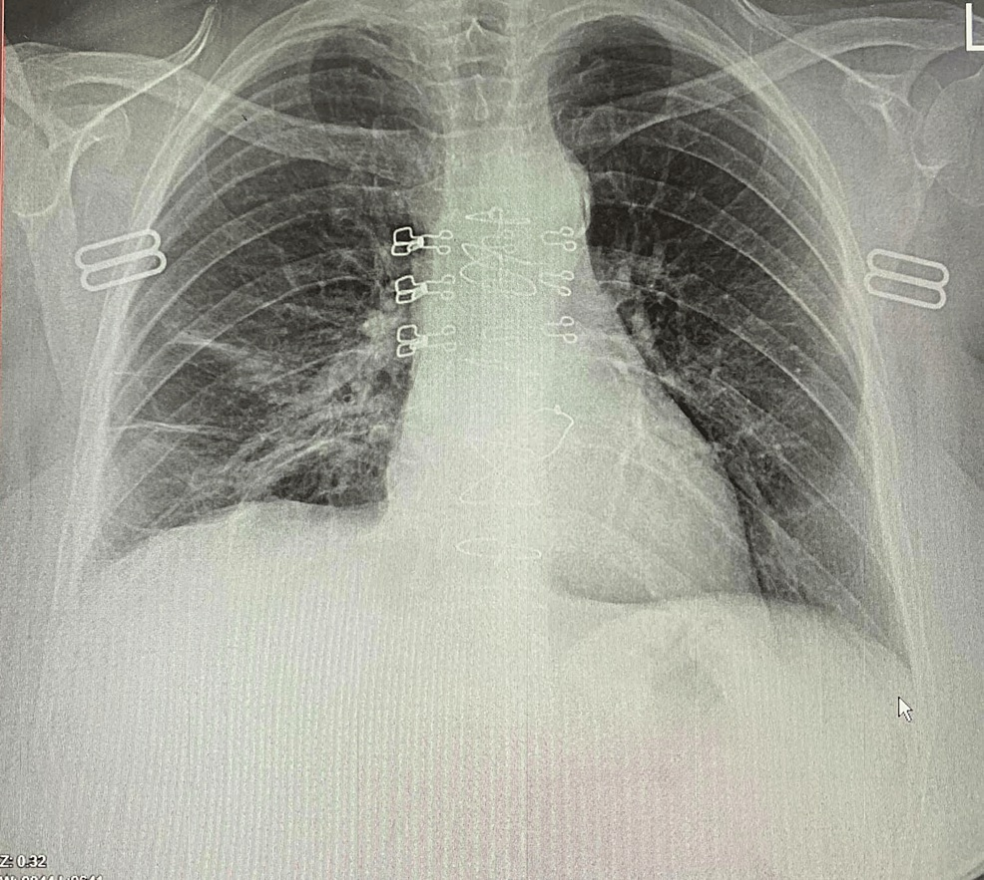
Chest X-ray of the patient on follow-up

The patient was diagnosed with post-cardiac injury syndrome and was treated with levofloxacin, ceftazidime, colchicine, 40 mg of prednisone daily, and other supportive drugs. The pigtail catheter was removed on the third day after admission, and six days later, the patient was discharged in good general health. The patient had a resolved pericardial and pleural effusion at the one-month follow-up visit and a normal range of inflammatory markers (Figure 3).

## Discussion

PCIS is characterized by pericarditis and pleuropericardial effusions that are a consequence of a heart injury [3]. The diagnosis of this condition is made easier by the PCIS clinical criteria, requiring the fulfillment of at least two of the five elements, which include the presence of a fever without a secondary reason, chest pain attacks (pericarditic or pleuritic), audible pericardial or pleural friction rub, pericardial effusion evidence, and with/or without pleural effusion in the presence of increased CRP [4]. Our patient was first identified as having pneumonia, but its clinical features, including fever, pericardial effusion, exudative pleural effusion, increased CRP, and chest pain, have been linked to PCIS. PCIS was detected in 1.7% of all open-heart surgery patients in a registry of 28,761 patients. After aortic surgery, mitral valve replacement, and replacement of the aortic valve, PCIS symptoms were present in the majority of patients, with aortic surgery having the highest frequency [5]. Fourteen point five percent (14.5%) of 822 individuals who underwent valve replacement surgery in another study experienced PCIS [6].

Younger age, female sex, summer season, kind of operation, history of corticosteroid use (current or in the past), prior history of pericarditis, lower body mass index (BMI), and some anesthesia have all been recognized as risk factors for PCIS and are generally agreed upon in the literature [7]. It's interesting to note that one of the systematic reviews that looked into PCIS risk factors found a total of seven studies with a summary indicating that each of inflammation, bleeding during the preoperative period, and blood coagulation may play a role in pathogenesis, suggesting multiple factors that contribute to the syndrome [6].

According to Dressler's hypothesis, myocardial necrosis, pericarditis, and pleuropericardial effusions result from an antibody-mediated hypersensitivity reaction following myocardial infarction in PCIS patients [3]. Due to its clinical features, which include symptoms like fever, development of anti-myocardial antibodies, activation of the complement cascade, numerous relapses, and having a particular incubation time, some articles have recently regarded it to be an autoimmune disease. Individual susceptibility also contributes considerably to this inflammatory process [2]. All of this supports the second-hit theory, which suggests that autoantibodies were created previously and sparked by heart injury [2].

In our case, the patient was female and had a chronic inflammatory condition that had been treated with prednisone in the past, which may have contributed to the development of PCIS. All of these factors might suggest that the syndrome is a combination of different symptoms caused by numerous etiological factors. Studying these risk elements could help clarify the pathophysiology of the condition and identify people who are at high risk.

## Conclusions

We suggest that there might be a possible reciprocal link between PCIS and SLE. We describe a case of pericarditis, pericardial effusion, and pleural effusion that may have been brought on by PCIS in conjunction with recent cardiac damage and an underlying chronic disease, primarily an autoimmune disorder. Due to the lack of data on the etiopathogenesis of PCIS, it is essential to describe all cases with various presentations and risk factors in the hopes of better understanding this pathogenesis.
